# Design Bioethics: A Theoretical Framework and Argument for Innovation in Bioethics Research

**DOI:** 10.1080/15265161.2020.1863508

**Published:** 2021-01-27

**Authors:** Gabriela Pavarini, Robyn McMillan, Abigail Robinson, Ilina Singh

**Affiliations:** a University of Oxford; b Abertay University

**Keywords:** Design bioethics, empirical bioethics, epistemology, games, videogames, young people

## Abstract

Empirical research in bioethics has developed rapidly over the past decade, but has largely eschewed the use of technology-driven methodologies. We propose “design bioethics” as an area of conjoined theoretical and methodological innovation in the field, working across bioethics, health sciences and human-centred technological design. We demonstrate the potential of digital tools, particularly purpose-built digital games, to align with theoretical frameworks in bioethics for empirical research, integrating context, narrative and embodiment in moral decision-making. Purpose-built digital tools can engender situated engagement with bioethical questions; can achieve such engagement at scale; and can access groups traditionally under-represented in bioethics research and theory. If developed and used with appropriate rigor, tools motivated by “design bioethics” could offer unique insights into new and familiar normative and empirical issues in the field.

## INTRODUCTION

There is growing recognition that an understanding of social context and public attitudes is valuable in bioethics (Dawson 2013; Ives 2008; Kon 2009). This value, however, is dependent on *how* this understanding is achieved. Empirical tools can help researchers describe and understand the complex nuances of morally relevant phenomena, but the field still grapples with the question of what constitutes “good” and “appropriate” empirical method in ethics (Ives and Draper 2009; Mertz et al. 2014). Moreover, there is growing understanding that researchers need to be critical and reflective about their methodological choices, as these will inevitably limit and bias perception and interpretation (Singh 2016).

Perhaps because empirical research came late to bioethics, bioethicists largely uncritically adapt empirical methodologies from other disciplines; e.g. interviews, surveys; behavioral experiments; neuroimaging data. Adaptation works reasonably well, as long as researchers have been trained to critically select and apply these tools. Too often, however, researchers do not make explicit how theoretical and/or epistemological commitments shape methodological choices (Singh 2016)—for example, if one is committed to a view of personal agency as an experience derived through processes of human interaction, then one is likely to choose research and analytic approaches that reflect this. One might, for instance, film interactions and conduct a discourse analysis to generate a grounded account of personal agency and threats thereto.

Another way forward in bioethics research is to *design* tools that explicitly integrate (or test) key theoretical commitments, and apply these to particular problems. This more creative approach is found in several methods used in bioethics research, including case-based moral dilemmas such as the trolley problem, as well as in newer approaches such as the contrastive vignette method e.g. presenting participants with different versions of short scenarios where protagonists face a moral dilemma and choose a course of action (Burstin, Doughtie, and Raphaeli 1980). Most of these designed tools are not exclusive to bioethics, nor did they originate in bioethics; however, they have been significantly adapted to be fit for purpose in bioethics. We see such tools as precursors of a category of purpose-built, technology-driven research tools that we call “design bioethics.”

## DESIGN BIOETHICS

We define design bioethics as the design and use of purpose-built, engineered tools for bioethics research, education and engagement. It is widely accepted that theoretical frameworks and methodological choices must go hand-in-hand: the choice of method involves an epistemological frame, which allows one to see and interpret a given phenomenon through a particular angle (Singh 2016). Design bioethics enhances researchers’ ability to meet theoretical and epistemological commitments, by offering methodological choice, control and flexibility. Digital technologies, including virtual and augmented reality; artificial intelligence, animation tools, wearable gaming, and holographic technologies, offer a range of novel features that can be harnessed for empirical research. Virtual reality, for example, allows for the illusion of being immersed in an alternative scenario or vividly belonging in another body (Slater et al. 2010), which has been used to study empathy and perspective taking (Peck et al. 2013).

When designing an empirical tool, researchers must consider the extent to which these different features are relevant to their adopted theoretical frameworks, as well as their chosen research questions. Considerations around the need for scale and reach, and cost-effectiveness, are also important. As such, design bioethics does not commit itself to a particular theoretical framework. Instead, it argues for critical, reflective and creative design of digital empirical tools, which align with the theoretical and epistemological commitments researchers bring to the table, as well as practical considerations.

The use of purposely designed digital tools in bioethics is currently scarce. However, educators have previously used digital games to teach ethics (Schrier 2015), and moral psychologists have digitalized, or created virtual reality versions of classic moral dilemmas, such as the trolley problem, for research (Francis et al. 2016, 2017; Navarrete et al. 2012; Pan and Slater 2011; Patil et al. 2014). More recently, a purely text-based game based on the movie Interstellar was created to investigate moral choices and inclinations (Pereira Santos, Khan, and Markopoulos 2018). In addition, a number of commercial games, both within the AAA and indie sectors, explore bioethical themes, including human enhancement and unregulated technology (BioShock by Irrational Games 2007; Deus Ex: Human Revolution by Square Enix 2011), AI in mental healthcare (Eliza by Zachtronics 2019) and eugenics (Fallout by Black Isle Studios 1997).

**Figure 1.**
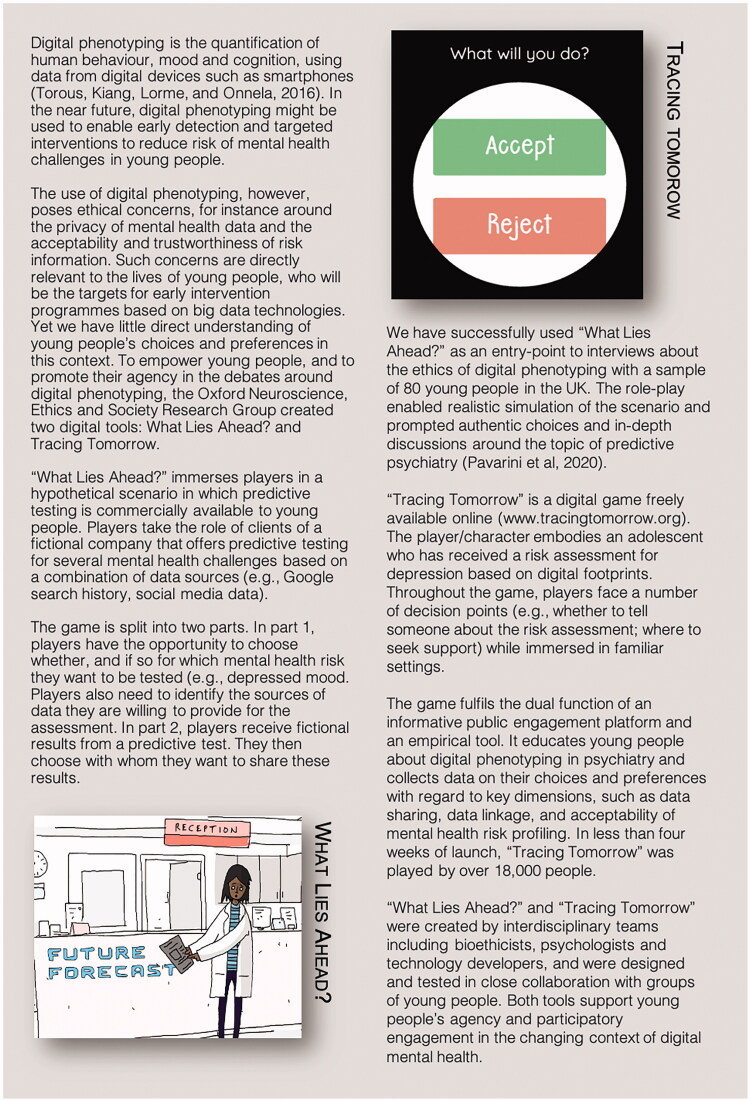
Using digital tools to investigate adolescents’ perspectives on the ethics of digital phenotyping for mental health.

If digital tools have already been used to investigate moral choices; to educate users in ethics debates; and more subtly to explore bioethical territory, then arguably it should be possible to design purpose-built game environments to conduct empirical research on specific bioethical research questions. As a proof of concept, our research group has created two digital tools for empirical ethics: a digital role-play scenario and a game, both of which focus on ethical issues surrounding the use of digital footprints in mental health risk assessments. These tools are described in Figure 1, as examples of the types of questions that might be investigated using bespoke digital tools.

Any purpose-built tool for bioethics research will require a theoretical scaffolding to guide its design, thereby enabling a kind of ontological reflection and transparency in method. In the following sections we highlight the ways in which digital tools can reproduce key theoretical commitments in bioethics, such that empirical research can be conducted through (not alongside) those commitments. Our focus is on context, narrativity and embodiment. These are not the only potentially useful features offered by digital tools; however, a comprehensive discussion of all potential features is beyond the scope of this paper. Our goal is to illustrate the opportunity for design bioethics—the chance to leverage technological advances at the interface of engineering, design and computing to build theoretically relevant and reliable tools for empirical bioethics research.

## THE IMPORTANCE OF LIVED EXPERIENCE IN CONTEXT

Whether, or to what extent, context should matter in the analysis of ethical problems has been a central tension between normative and empirical approaches in bioethics. As we have outlined elsewhere (Pavarini and Singh 2018), we have found pragmatism to usefully articulate how and why context should matter to bioethical investigation. Dewey's (1998) pragmatism proposes that context is crucial for moral decision-making because one cannot conceptualize the moral self as separate from daily experience. Following this framework, the tools of empirical ethics should aim to capture lived experiences of ethical values and concepts.

Although pragmatism is significant, it is not the only approach in ethics in which lived experience is considered to be ethically and morally relevant. Other intellectual communities within bioethics have made similar arguments: feminist bioethics conceptualizes moral choices as embedded in relationships and social context (Gilligan 1982; Walker 1998), while moral particularists hold that the moral status of an action is defined by relevant features of a particular context, analysis of which should inform a normative account of action (Hooker and Little 2000; Arras 1991). Lived experience as source-material for ethical norms also occupies an increasingly central place in contemporary bioethics (Dawson 2013; Ives 2008; Kon 2009). While there are important distinctions among scholars who promote the importance of context and experience in bioethics, collectively such perspectives have been positioned as a departure from principlism, which is seen to privilege universal moral values and guiding rules over individual situations and the judgments they call for (Beauchamp and Childress 2013; Engelhardt 1986; Veatch 1981).

The acknowledgement that ethics and morality are embedded in actions and social context poses an epistemic challenge for researchers: “experience” or “situated choices” are difficult to access empirically. Surveys and interviews about moral attitudes and values are the most common methods in the field (Wangmo et al. 2018), but while these can be illuminating of research questions they are also limiting, because the scenarios they provide participants to engage with are necessarily distal in time and place to an actual situation. We propose that digital tools such as games and VR scenarios can provide a more proximate “real world” solution, since they allow for judgments and choices to be embedded in (designed) context and social interactions (Gee 2013; Sicart 2013). For example, in an episode of *Life is Strange* (Dontnod Entertainment 2015), players embody a character, Kate, who witnesses a friend holding a knife at the school toilet. Kate is later confronted by the school principal, and is given the opportunity to disclose the truth about what she has seen, or to hide it. Even though *Life is Strange* was not designed as an empirical tool, players’ choices might reveal something about how they balance honesty, safety and loyalty, in a concrete case scenario.

### Is Gaming Context an Appropriate Model for Real-World Choices?

Although we argue that games are more proximate to “real world” experiences, a number of potential limitations must be pointed out. A first and fundamental question refers to whether the context created in a game or digital scenario is an appropriate model for “real world” context. A corollary question is whether “real world” is indeed what such scenario should try to approximate in order for research results to be valid. For example, Fallout 3: Quest Oasis (Bethesda Game Studios 2008) confronts players with the decision of whether to intentionally end another’s life for compassionate reasons. The scenario itself is set in fantasy: the target character who pleads with the player character to grant him the right to die is a talking tree, who had been a human but became rooted due to a virus. Can we assume that decisions made in this metaphorical scenario, and within the constraints of the game, would reflect a player’s moral values and decision-making in real right-to-die scenarios? Empirical research should investigate the extent to which metaphorical scenarios might constrain research validity.

Another more practical limitation refers to the fact that participants/players might lack relevant information required to make the decision, or misunderstand the information provided. In an interview setting, misunderstandings can be easily identified and resolved by the researcher. However, if a game is used in an online setting, or participants interact with it independently, potential errors might go unnoticed. As Sinnott-Armstrong (2018) points out, accurate background knowledge about the question at hand is a fundamental step toward making a good moral decision. For instance, for decisions surrounding predictive genetic testing, accurate information about a test’s predictive value and data handling policies would enable more informed decision-making.

In order to maximize the chances that misunderstandings are identified and corrected, researchers must subject their games to extensive piloting, using methods such as “think out-loud” where participants are asked to verbalize any thoughts they have while playing the game (Gaboury and Ladouceur 1989). It is also possible to build in tools such as chatbots within a game, which can test whether participants are aware of certain pieces of information, correct misunderstandings, and provide additional information. Finally, purpose-built games also allow researchers to provide groups of participants with different background information to test its effect on ethical decision-making.

Finally, even though context can de-bias decisions, the choices that designers or investigators make about what context to include or exclude can be a source of bias. For instance, the context of decision-making might be leading, or too specific to generalize. Psychological studies have suggested that a number of “morally irrelevant” features of scenarios, such as the order in which events are presented or the wording (e.g. actions framed in terms of “killing” vs. “saving”) can sway people’s judgments and intuitions (Petrinovich and O’Neill 1996). This problem can be partially addressed leveraging statistical power: because digital tools afford large-scale data collection, it is possible to vary contextual factors across participants. For example, Awad et al. (2018) developed an online game to investigate moral preferences around dilemmas faced by autonomous vehicles during unavoidable accidents. The game presented participants with numerous versions of trolley-type cases, varying thirteen different factors including characters’ age, gender and social status. The possibility of modifying narratives, characters and choices enabled thoughtful design of the game as a valid empirical research tool. By including numerous contextual variations, games can control for bias; and by randomizing exposure to different contextual clues, researchers can ascertain the relative contribution of each factor on decision-making.

## NARRATIVE AS CONTEXT FOR AUTHENTIC DECISION-MAKING

One might argue that design bioethics is simply proposing digitalized versions of tools that are already available in a bioethicists’ toolkit; for instance, games might be seen as a digital version of a contrastive vignette technique. Even though this is partially true for brief digital scenarios, more complex scenarios and games have unique features that distinguish them from traditional vignette methods. One such feature is narrative continuity.

Contrastive vignette studies often rely on static hypothetical scenarios, typically presented to participants in text or image, often in the third person. As with other two dimensional story-based scenarios, contrastive vignettes are limited in the emotional and contextual grounding they can provide; a participant is embedded in a single moral dilemma (or a series of single moral dilemmas) and does not have a stake in the outcome of a story powered by their decisions. In a game, participants can embody a character (first-hand) that is embedded in an ongoing series of moral choices that make up an overarching narrative. Through the use of branching narratives within a game (Adams 2010), players’ moral choices affect how the storyline develops both in the short-term and in the long-term, giving them the chance to experience potential consequences of their actions, and make choices that build on previous ones (Riedl and Young 2006). In other words, players are made responsible for their choices as part of a developing story, which arguably encourages more authentic responses in comparison to decontextualized vignettes. Players might also be given the possibility of re-winding a scenario to make an alternative choice e.g. after learning new facts, and explore a different branching narrative.

The importance of narrative as a context for moral experience and conceptualization of the good is at the core of work aligned with narrative bioethics (Charon and Montello 2002; Frank 1997; Jensen and Mattingly 2009; Nelson 1997). Virtue-based accounts of ethics also hold that the narrative form of human life provides a basis for the virtues (MacIntyre 1981). For Taylor, the sweep of history itself offers a “source” for moral self-understanding: “because we have to determine our place in relation to the good, therefore we cannot be without an orientation to it, and hence must see our life in story” (Taylor, 1989, 51–52). Empirical evidence for the importance of narrative for ethical reflection, moral identity development and the transmission of moral values is also provided by studies in the field of moral psychology (Krettenauer and Mosleh 2013; Matsuba and Walker 2005; Tappan and Brown 1989). Therefore, interactive narrative games offer a unique tool for researchers aligned with these theoretical framings of moral experience.

### Do Gaming Narratives Do Justice to the Richness of Real-World Narratives?

One could argue that narratives within a game are qualitatively different from those that emerge in real life encounters: aspects of the narrative in the game, and options for its development, are pre-programmed and thereby fail to capture subjective and intersubjective processes of narrative construction. Further, the important interactive element that is present in interviews and participant observations is lost in a game setting. These are fair objections. However, even in the most structured games, players do not passively surrender to the storyline. They actively engage with the content and role-playing, and construct a personal narrative, albeit within the parameters of game ontology (Brathwaite and Sharp 2010). These parameters are inherent to the game; other important limitations are cost and imagination.

Furthermore, even though games might lack interactive elements, by including two or more players whose actions affect one another, a researcher is able to collect data within social interactions. In fact, some gaming environments that afford high levels of flexibility, autonomy and interactivity, which would suit researchers wishing to investigate naturalistic interactions. For example, in Second Life (2009) players create virtual representations of themselves through which they explore the environment, socialize, shop, buy properties, and so forth, thereby blurring the boundaries between reality and fantasy (Schechtman 2012; Spurgin 2009). These environments allow for surveys, interviews, and detailed, in-depth analysis of everyday life and practice (Guo and Barnes 2011; Houliez and Gamble 2013; Partala 2011). Ethnographers who have immersed themselves in these settings describe Second Life not as a fictional tale detached from reality, but rather as a “profoundly human” experience (Boellstorff 2015, 5; also see Nardi 2010 for a similar account of World of Warcraft). The virtual nature of these settings affords participants a high level of privacy where they are able to talk openly about sensitive moral issues. Researchers also have the choice of manipulating the environment to create morally relevant dilemmas for players, rather than simply engaging in observation of natural behavior.

Both highly structured games and flexible gaming environments can be used exclusively or integrated into a larger research protocol (e.g. providing an entry point into a real-life qualitative interview or survey), depending on the researchers’ goals. Furthermore, given that morality is not unidimensional (Gibbs 2019; Lapsley and Narvaez 2004), it is important that researchers define what moral dimension they are interested in assessing through games or otherwise. Possibilities include time-slice moral choices or behavior; dispositional moral orientation or identity; moral emotions; judgments of other people’s actions; attitudes toward abstract moral concepts; and moral reasoning.

Some might wonder, however, why a researcher might construct fictional scenarios or engage with virtual settings, when a number of alternative methods allow them to investigate these constructs in actual, “real-life” narratives and environments. Ethnography or other observational methods, for example, allow for rich descriptions of people’s moral experiences and judgments as they unfold in real life (Kleinman 1999; Parker 2007). A core limitation, however, is that these methods do not apply to every question at hand. Critically, many of the questions that interest empirical bioethicists concern implications of new technologies that have not yet arrived or been made accessible (e.g. artificial wombs; Kendal 2017). In other cases, the technology is already available, but there is an interest in understanding the ethical perspectives of *potential* users/stakeholders (e.g. females’ views on egg freezing, Baldwin et al. 2014). In yet other cases, there is an interest in learning about how ordinary people, with no direct involvement with the ethical issue of interest, think about certain questions (e.g. general public’s views on gene editing for muscular dystrophy). In all these cases, the attitudes sought refer to scenarios that are not (yet) real. Exploring the intricacies of these “imagined realities” requires tools that allow for realistic simulations of moral scenarios, of which digital games are a prominent example.

It is also worth noting that when research questions and other considerations call for direct observation of “real-life” moral experience rather than simulated scenarios, this can also be achieved through purpose-build digital tools. For instance, psychology has pioneered innovative digital methods that get close to people’s moral choices in day-to-day life. This includes unobtrusive recordings of morally relevant dialogues in everyday life via participants’ mobile phones (Bollich et al. 2016) and momentary assessments where participants are prompted to report on real-life moral experiences every time a mobile signal arrives (Hofmann et al. 2014; Pavarini and Singh 2018). These methods are accessible and scalable, and certainly have much to add to a more “situated” or embodied bioethics.

The process of creating a digital game for research is, however, costly and time-consuming. Could the same effect be achieved using a text, paper-based tool, along the lines of choose-your-own-adventure fiction book? Researchers have argued that literary fiction offers valuable simulative experiences of social scenarios (Mar and Oatley 2008), allowing readers to immerse themselves into a rich narrative, and take on different roles and perspectives (Kidd and Castano 2013). In choose-your-own-adventure books, players take on an active role shaping the storyline, much like in a digital game. What is the added value of digitalizing the narrative? In the next section we discuss the importance of embodiment and immersion, which distinguish digital games from written fiction.

## EMBODIMENT AND DECISION-MAKING

We understand moral decision-making to be an embodied process, which involves physical, affective and cognitive components (Tappan 1990; Walker 1998). Grounded accounts in philosophy of mind and contemporary cognitive science also emphasize that moral-decision making should be understood with reference to the social and embodied context where it is situated (Laham and Kelly 2018; Prinz 2009). In line with this view, games provide a research platform that allows moral decision-making to be grounded in situated scenarios that elicit high-fidelity cognitive and emotional responses (Gee 2008; Vorderer and Bryant 2006). In tandem with narrative presence (e.g. personal engagement with a storyline within a game), digital tools can produce simulations that mimic unmediated sensory experiences. This gives rise to unparalleled feelings of physical presence—that is, the illusion of being present in the virtual setting, and emotional presence—the experience of real affective responses to the game (Ryan, Rigby, and Przybylski 2006).

Digital role-play, for instance, has the potential to offer players either the realistic illusion of inhabiting an avatar’s body (Fox and Ahn 2013), or the impression that their own body occupies the virtual environment conveyed by the game (Tamborini and Skalski 2006). This is particularly true for virtual reality experiences (Ahn et al. 2016; Ahn, Le, and Bailenson 2013). Taken together, these elements create what has been referred to as “illusion of nonmediation” (Lombard and Ditton 2006) between the user and the digital context, generating a highly engaging, immersive experience. Indeed, the highly emotionally arousing and action-focused nature of digital games have been shown to result in moral choices that differ from choices made using text-based methods (Francis et al. 2016, 2017).

There is extensive evidence, for example, that digital games engage moral thoughts and emotions in ways that are akin to real-life scenarios. Even brief laboratory exposure to graphically violent video games increases physiological arousal, aggressive thoughts and feelings, and antisocial behavior (see Anderson et al. 2010; Ferguson 2015 for meta-analytic reviews). Similarly, playing prosocial video games increases prosocial thoughts, positive emotions and helping behavior (see Greitemeyer and Mügge 2014 for a meta-analysis). These effects also go beyond the gaming context. For example, there is correlational, longitudinal and experimental evidence that teenagers who play prosocial digital games (e.g. Super Mario Sunshine, Chibi Robo) or games involving civic experiences (e.g. Guild Wars 2) are more likely to act prosocially in everyday life than teenagers who do not have the same experience (Gentile et al. 2009; Lenhart et al. 2008). In sum, games modulate real-life ethically-relevant thoughts, emotions and behaviors in both the short and long-term.

Players feel psychologically connected to their avatar and react with guilt and shame when their player character behaves immorally (Grizzard et al. 2014; Hartmann, Toz, and Brandon 2010; Mahood and Hanus 2017). Players also express distress when their character is the victim of morally reprehensible acts (Gabriels, Poels, and Braeckman 2014; Wolfendale 2007). In an iconic case from 1992, a woman reported symptoms of posttraumatic stress after her online avatar had been sexually assaulted at LambdaMOO, a text-based online virtual reality system to which multiple players are connected simultaneously (Dibbell 1993). In psychology, the Cyberball paradigm (Williams, Cheung, and Choi 2000), whereby research participants are ostensibly ignored during an online ball-tossing game, is also known to induce strong negative emotions (Chow, Tiedens, and Govan 2008) and biological stress responses (Slavich et al. 2010).

Despite these encouraging findings, a note of caution is warranted. First, the potential for immersion that digital games and scenarios afford is neither fixed nor homogenous. Technical elements such as the type of visual display and subjective features such as attention and personal connection to the storyline can significantly alter a game’s potential to induce a sense of presence (Cummings and Bailenson 2016; Shin 2018). Furthermore, even though novel tools such as motion-controlled systems (e.g. Nintendo Wii) and augmented reality afford actual physical presence, most games only induce *feelings* of physical presence. Given that physical features are significant to embodiment and experience (Scully 2008), it remains to be tested whether the *illusion* of presence and *actual* physical changes have differential effects on moral reflection and decision-making.

### Do Games Elicit Authentic Moral Choices?

An important question within design bioethics, which applies particularly to games and role-play tools, refers to authenticity. Despite being an immersive experience, one might argue that players’ moral character and choices within game contexts are unlikely to match those in offline settings. Would it be possible that the virtual nature of games and the “plasticity of avatars” (Bailenson and Beall 2006) allow for a temporary suspension of the player’s moral conscience, giving them the opportunity to make choices they would not dare perform in real life? Empirical data do not necessarily support this hypothesis. To the contrary, there is evidence that individuals typically express themselves honestly and authentically in virtual environments (Bargh, McKenna, and Fitzsimons 2002). In gaming contexts, even though players tend to select avatars that are *physically* closer to their ideal selves (see Sibilla and Mancini 2018 for a review), they prefer avatars and characters that reflect their moral dispositions (Delhove and Greitemeyer 2020; Ewell et al. 2016). Critically, in several empirical studies using game players, most players reported that their gaming decisions were analogous to how they would behave in real life, and these choices were indeed predicted by their moral values and intuitions as measured by self-reports (Boyan, Grizzard, and Bowman 2015; Krcmar and Cingel 2016; Pereira Santos, Khan, and Markopoulos 2018; Weaver and Lewis 2012). In sum, in a game designed for research, virtual moral choices are unlikely to deviate too far from those in real-life settings. However, the reliability of players’ moral choices, relative to “real life” choices, is an aspect that would need to be tested in a pilot phase.

A number of other measures might be taken to maximize the chances that players respond authentically. As Schrier (2019) suggests, constructing realistic dilemmas and including quotidian elements into the narrative can create the feeling that the game is realistic and this way elicit more authentic choices. Researchers can also tell participants upfront that the game or digital role-play is used for research, explicitly asking them to make choices as if it was a real experience and (if applicable) clarifying that their answers are anonymous. Another way of eliciting more genuine choices is by framing the game as an opportunity for players to learn about themselves and offering insights into their choices (e.g. where they stand in comparison to previous players) at the end of game playing.

Finally, it is important to note that it is possible to design a game that purposely manipulates players to make moral choices that they would not make outside the game context. Numerous commercial and noncommercial games explicitly elicit antisocial behavior. For example, in Fake News (ISL Gaming 2017), players are incentivised to post deliberate pieces of disinformation on social media; in violent games such as Counter-Strike (Valve Software 2000) players perpetrate acts of terror including bombing and assassination. In other games, morally relevant choices might be biased by the overall goal assigned to the player. For instance, in The Uber Game (2017) players embody an Uber driver and are assigned the single goal of earning as much capital as they can, which might lead users to overlook relationship goals or break the law.

As discussed earlier, researchers need to be aware of the potential and the limitations of the tools they use for research, and the epistemological frame (and biases therein) created by their methodology. If a game is used to assess moral decision-making on a “real world” challenge, it is important that the players’ moral choices can be treated as authentic; therefore players should not be incentivised to make a particular decision; e.g. moral choices should not drive success or failure in the game. However, if the aim of the research game is to see, for example, how far players will go in an immoral direction for perceived rewards (as in the obedience experiments by Stanley Milgram 1963), then a game that purposely manipulates players might be the right choice. Incentives might also be used to motivate players, as typically done in laboratory experiments in economics (Camerer et al. 2016). Researchers can work in collaboration with game designers and experts in human-computer interaction (Barr, Noble, and Biddle 2007) to create digital environments that are naturalistic and conducive to authentic game playing.

## THE EMPIRICAL ADVANTAGE OF a DESIGN BIOETHICS RESEARCH TOOL

In addition to offering the potential for contextualization, narrative presence and embodiment, games and other digital tools offer practical advantages for bioethics research in comparison to traditional methods. First, games have been shown to improve participant engagement and motivation (Cordova and Lepper 1996), which arguably increases the richness, quality and authenticity of collected data. Furthermore, digital devices are popular and accessible medium: design bioethics tools can be hosted on accessible online platforms and marketed to thousands, or even millions of individuals, across sociocultural and geographical boundaries. Thus, digital tools offer the possibility of efficiently conducting research at scale and including traditionally under-represented voices in bioethics (e.g. young people), increasing robustness, representativeness, and generalisability of results. This is relevant in the current context, where most bioethics research involves small sample sizes (Wangmo et al. 2018), but results are often taken as indicative of societal preferences and inform the work of policymakers and practitioners. It is important to note, however, the challenges presented by the “digital divide,” which describes inequalities in access to data, internet and digital tools, and digital skills (Gonzales 2016). Such challenges must be acknowledged at the start of the project, as some solutions can be designed into the tool. For example, in areas where data is limited or the network is slow, a low-tech game or SMS chatbot tool might be more effective than a graphically demanding tool.

In addition to the possibility of engaging large numbers of participants, digital platforms allow researchers to collect data on several peripheral aspects of moral decision-making. For example, researchers can track the duration of a moral decision, physical movements and gazing patterns, interactions with specific widgets or other players, typing patterns, geographical location, mouse switches between options (e.g. indicating ambiguity in decision-making) etc. Researchers can also triangulate these different data sources to obtain a rich and nuanced understanding of participants’ decision-making, and an understanding of where peripheral elements are relevant. For example, virtual reality scenarios have been used to investigate whether physical distance from a particular scenario can influence moral engagement (Petras, Ten Oever, and Jansma 2015).

Depending on the nature of the data collected, researchers might also choose to make the dataset open access for secondary analyses. This could be either a permanent dataset downloaded after reaching a given sample size, or a changing dataset, which is constantly updated the more participants engage in game playing. If digital tools become more common in bioethics, a unified platform to host games, tools and datasets could be created, which would certainly provide a step-change in our ability to generalize and maximize the impact of empirical results. These datasets might include quantitative data (e.g. choices during gameplay) as well as qualitative data on participants’ motivations, aspirations etc. collected, for instance, via a chat function. The sharing of large datasets within bioethics allows for robust empirical analyses and conclusions, which can then inform normative reasoning.

## ETHICAL AND PRACTICAL CONSIDERATIONS

Alongside the benefits of adopting a design bioethics approach, a number of practical limitations are worth considering. By using a digital tool that participants can access and interact with independently, researchers have less control over who is included in the sample, and less certainty that the answers obtained are genuine e.g. that participants are not misrepresenting themselves. For games in particular, even if some measures are taken to prevent individuals for playing multiple times (e.g. placing cookies that prevent the game from being played twice on the same device), researchers cannot fully guarantee that each data point is independent. Finally, tech-savvy, game-enthusiast individuals, who have access to fast broadband, might be overly represented in the sample.

There are also a number of broader ethical considerations, particularly from the participants’ standpoint. For instance, there is growing recognition that digital media forms—the Internet, smartphones, video games—can be addictive and time-consuming (Duke and Montag 2017) and that game addiction leads to poor mental health outcomes in young people (Wang, Sheng, and Wang 2019). Researchers must assess whether their digital tool has the potential to trigger addictive use and take measures to minimize this risk (e.g. adjusting rewards, adding screens that encourage players to take down time).

The immersive nature of digital scenarios, combined with the sensitivity of some bioethical topics, might also pose a risk to the emotional well-being of some participants. Furthermore, the realistic nature of futuristic scenarios within a game (e.g. gene editing for human enhancement) might induce false beliefs about what is currently done or available. The potential risk of creating discomfort or misbeliefs must be carefully assessed before a digital game is disseminated to the target audience. Researchers must seek to minimize such risks, for instance by adding in-game measures of mood and implementing clear debriefing procedures.

If data are hosted online, and transferred via online networks, a number of extra precautions around safety, privacy and confidentiality are warranted (Emery 2014; Martinez-Martin et al. 2018). Furthermore, if players can access the design bioethics tool independently, researchers will be limited in their ability to obtain robust informed consent and assent, including verifying participants’ age, and limited in their ability to implement comprehensive debrief procedures (Eynon, Fry, and Schroeder 2012; Keller and Lee 2003). Before embarking on any digital bioethics project, researchers must consider these limitations and reflect on ways to circumvent them.

## CONCLUSION

We have argued that bioethics research can be significantly improved when our tools harness novel technologies in reflexive ways that align with explicit theoretical commitments. We have called this approach “design bioethics.” As part of our argument for this approach, we have illustrated the ways in which digital games (as an example of a designed digital tool) enact key theoretical commitments found across empirical bioethics: context, embodiment, and narrativity. A design bioethics approach allows researchers to consider how designed tools can enact their own theoretical commitments and also respond to practical considerations (e.g. whether the tool must be scalable). The approach invites critical, reflexive and creative design of empirical tools, attending to theoretical, epistemological and practical considerations.

Clearly design bioethics is an inherently interdisciplinary endeavor, which requires an admittedly difficult, but rewarding process of distilling intellectual interests, values and commitments into the ontological build of a purpose-built research tool. This process ideally involves experts in e.g. AI, design engineering, computer programming or human-computer interaction, who will ensure the quality of participant-platform interaction elements, including engagement, enjoyment, information input/output (e.g. visual displays, motion), and platform contents (e.g. narrative) (Caroux et al. 2015; Sánchez et al. 2012). Researchers’ expertise needs to ensure that research quality is prioritized; an AI platform designed as a research tool requires the same scrutiny as other methodological choices and empirical tools, and should be subject to the same quality indicators as, e.g. surveys and interview guides. A discussion of what these quality indicators should look like is beyond the scope of this paper, but have been discussed by others in the field (Mertz et al. 2014).

While a design bioethics approach can be used to investigate current moral and ethical scenarios across health and social care, we think it has particular promise for investigation of emerging biomedical and neuroscience technologies that fall outside current experience. Rapid advances in biology, medicine and technology including AI applications, cyber-body modifications, gene editing and others, require reflection on moral and ethical challenges that fall outside the here and now. Digital games, virtual reality, holographic designs and more can transport us into these imagined realities and set us on a journey of embodied ethical exploration. By immersing participants in these worlds, research may reveal a deeper truth about self-understanding, and about individual and societal attitudes, values and priorities. We anticipate that the results will yield more relevant, representative and trustworthy assessments of what ought to be done.
